# External beam focal boost radiotherapy to intraprostatic lesions in prostate cancer: a scoping review

**DOI:** 10.1016/j.phro.2026.100980

**Published:** 2026-04-25

**Authors:** Ying Liu, Shangbin Qin, Xueying Ren, Yun Bai, Xianshu Gao, Hongzhen Li, Mingwei Ma

**Affiliations:** a Department of Radiation Oncology, Peking University First Hospital, Beijing 100034, China; b Department of Radiation Oncology, Peking University People’s Hospital, Beijing 100044, China

**Keywords:** Prostate cancer, Radiotherapy/Radiation Therapy, External Beam Radiotherapy (EBRT), Simultaneous Integrated Boost (SIB), Intraprostatic lesions (IPLs), Dominant intraprostatic lesions (DILs)

## Abstract

**Background:**

Focal boost external beam radiation therapy (EBRT) delivers an escalated dose to intraprostatic lesions (IPLs) in prostate cancer. It is an emerging field characterized by highly heterogeneous practices.

**Materials and methods:**

This scoping review was conducted with adherence to the Preferred Reporting Items for Systematic Reviews and Meta-Analyses extension for Scoping Reviews guidelines. Eligible reports were reports detailing treatment methods and outcomes of focal boost EBRT in prostate cancer.

**Results:**

Forty-two studies were reviewed. Fractionation shifted over time from conventional to moderate and ultra hypofractionation. Patient populations were largely high-risk localized disease. Assuming α/β = 1.5, equivalent IPL boost dose in 2 Gy/fraction spanned 71.3–138 Gy in 32–45 fractions with conventional fractionation, 81.5–112.5 Gy in 15–25 fractions with moderate hypofractionation, and 96.4–196.4 Gy in 2–5 fractions with ultra hypofractionation. Multiparametric magnetic resonance imaging (MRI) was used for IPL delineation in 39 studies, and positron emission tomography/computed tomography (PET/CT) in five studies. Five-year biochemical disease-free survival and overall survival were: conventional fractionation 92–98.2% & 100%, moderate 75.2–96.7% & not reported, and ultra hypofractionation 93.2–100% & 86–100%. Median ≥ Grade 2 genitourinary/gastrointestinal toxicities were < 45% and ≥ Grade 3 < 10%.

**Conclusions:**

Focal boost EBRT appears feasible and may improve biochemical control without increasing toxicity, particularly in high-risk disease. However, optimal dose/fractionation, organ at risk constraints, and long-term survival benefits remain undefined. Large, preferably multicenter prospective studies involving several hundred patients, with standardized endpoints and comprehensive toxicity assessment are warranted.

## Highlights

• Focal boost external beam radiotherapy in prostate cancer has been reported in 42 studies.
• Doses to intraprostatic lesions varied from 1.88 Gy to 16 Gy per fraction.
• Five-year biochemical disease-free survival rates were 75.2–100%, and severe toxicities were uncommon, typically < 10%.

## Graphical abstract

**Figure:**
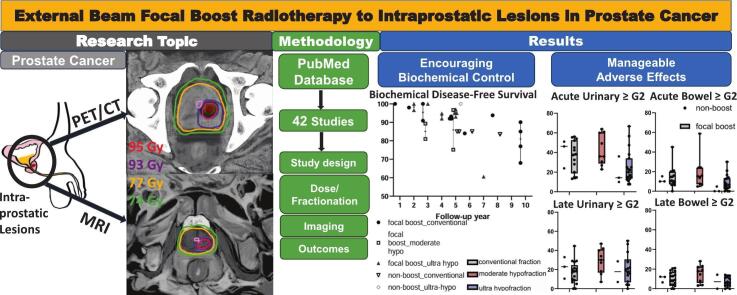

## 1 Introduction

Prostate cancer is one of the most common malignancies in men, with an incidence continuing to rise globally, particularly in parts of Africa, Asia, and Latin America [1]. The annual number of new cases is projected to increase from 1.4 million in 2020 to 2.9 million by 2040 [2]. For non-metastatic disease, especially in patients with a life expectancy of > 10 years, the National Comprehensive Cancer Network (NCCN) guidelines recommend radical radiotherapy as a key curative approach with outcomes comparable to surgery [3].

Recurrence after radiotherapy poses a significant challenge. Even in the high-dose era (75.6–86.4 Gy to the whole prostate), cumulative 8-year local recurrence rates remain 9.8% and 14.6% for intermediate- and high-risk patients [4]. Dose escalation has been shown to improve biochemical relapse-free survival [5], [6], [7], with each additional 1 Gy estimated to reduce recurrence risk by approximately 1.8% [8]. However, in conventional radiotherapy where a uniform dose is delivered to the whole prostate, further escalation is restricted due to the inevitably increased toxicity to nearby organs such as the urethra, bladder, and rectum [9], [10].

Several imaging-pathology correlation studies have found that most local recurrences of prostate cancer occurred within previously radiographically visible intraprostatic lesions (IPLs) [11], [12], [13]. This led to the concept of focal boost radiotherapy, which delivers an escalated dose to the IPLs or dominant intraprostatic lesions (DILs) in an attempt to improve local control while minimizing toxicity. Advances in intensity-modulated radiotherapy have meant that it is now possible to deliver simultaneous integrated boost (SIB) using external beam radiotherapy (EBRT), enabling precise “dose painting” within the prostate.

Focal dose escalation to imaging-defined IPLs has been explored clinically for over a decade, with variability in patient selection, imaging modalities, and treatment delivery. Several systematic reviews published in 2023 have summarized aspects of this field: Zhao et al. [14] summarized planning studies and clinical trials of focal boost using EBRT or brachytherapy, while Poon et al. [15] and Dornisch et al. [16] focused particularly on EBRT focal boost to magnetic resonance imaging (MRI)-defined IPLs. However, these reviews predate rapidly growing evidence, especially studies employing moderate/ultra hypofractionation and positron emission tomography/computed tomography (PET/CT)-guided IPL definition. To capture more recent evidence and fill the information gaps, a scoping review of published clinical studies on definitive EBRT-based focal boost treatment in newly diagnosed, non-metastatic prostate cancer was conducted.

## 2 Materials and methods

This scoping review was carried out using the guidelines outlined in the Preferred Reporting Items for Systematic Reviews and Meta-analyses (PRISMA) Extension for Scoping Reviews (PRISMA-ScR) [17].

A comprehensive PubMed search was performed without date cut-offs and last updated in April 2025. Studies were eligible if they enrolled patients with histologically confirmed prostate adenocarcinoma who received definitive-dose EBRT combined with a focal boost to IPLs (or all synonyms as defined in the search strategy (Supplementary Material A)). Exclusion criteria included the presence of distant metastases (M1 stage), recurrent disease after prior definitive therapy, boost delivery via brachytherapy, absence of toxicity or clinical outcome data, lack of full-text availability, or focal boosts targeting areas outside the IPLs. Additional eligible studies identified through the reference lists of articles included were also incorporated. The detailed study selection process and characteristics of studies included are presented in the Results section; in total, 42 studies comprising approximately 4800 patients were included.

Independent title and abstract screening, followed by full-text eligibility assessment, were conducted by two reviewers (Y.L., MBBS and X.R., MD). Discrepancies were resolved through discussion, under the guidance of a third reviewer (M.M., MD). Data extraction was performed by multiple reviewers (Y.L., MBBS, X.R., MD, S.Q., MD and M.M., MD) and cross-checked for accuracy.

## 3 Results

**Fig. 1:**
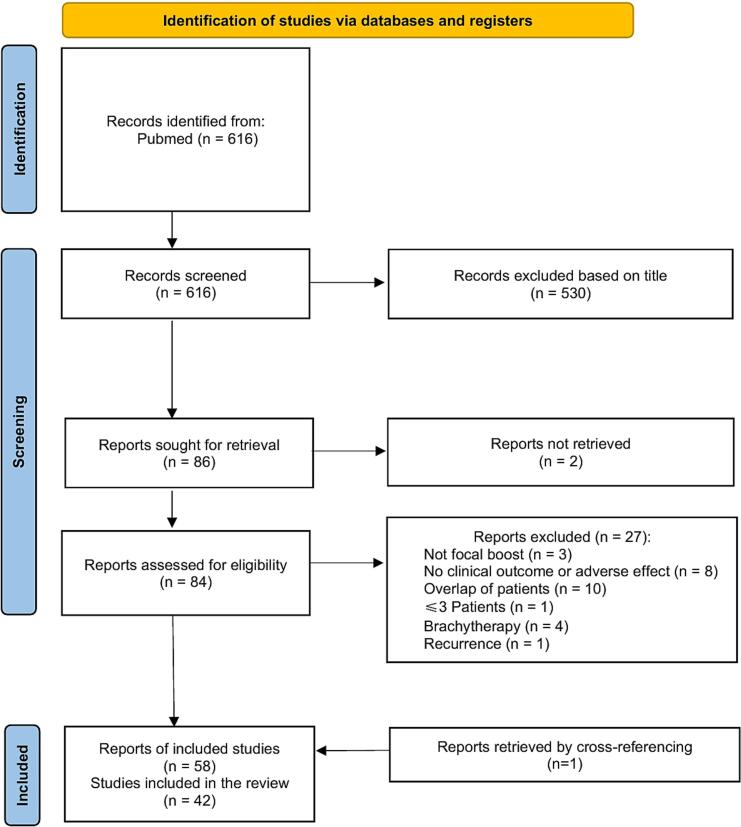
PRISMA flow diagram showing the study inclusion and exclusion process. A PubMed search identified 616 records. After title screening, 86 reports were retrieved for full-text review, of which two were unavailable. Following eligibility assessment, 27 reports were excluded for reasons including lack of focal boost (n = 3), no clinical outcome or toxicity data (n = 8), patient overlap (n = 10), ≤ 3 patients (n = 1), brachytherapy (n = 4), or recurrence (n = 1). One additional report was identified through cross-referencing. In total, 58 reports representing 42 unique studies were included in the review.

A total of 616 records were identified and assessed per predefined criteria. Fifty-seven reports met the inclusion criteria, and one additional report was identified through screening the reference lists of the included studies, yielding 58 reports corresponding to 42 unique studies (Fig. 1). The studies were classified by fractionation schemes, with 14 studies exclusive to conventional fractionation detailed in Table 1 [18], [19], [20], [21], [22], [23], [24], [25], [26], [27], [28], [29], [30], [31], [32], [33], [34], [35], [36], [37], [38], nine studies with moderate fractionation detailed in Table 2 [39], [40], [41], [42], [43], [44], [45], [46], [47], [48], [49], and 19 studies exclusive to ultra hypofractionation detailed in Table 3 [50], [51], [52], [53], [54], [55], [56], [57], [58], [59], [60], [61], [62], [63], [64], [65], [66], [67], [68], [69], [70], [71], [72], [73], [74], [75]. Prospective designs accounted for 50% (7/14) of conventional fractionation studies versus 89% in moderate (8/9) and ultra hypofractionation (17/19) studies. Temporal analysis revealed a transition in fractionation approaches. Among conventional fractionation studies, 12 of 14 were first reported before January 2023, while 11 of 19 ultra hypofractionation studies were published from January 2023 onwards.

**Table 1:** Overview of conventional-fractionation focal boost EBRT regimens.

**Study** **Author, Year**	**Study Type**	**Sample Size** **(high risk)**	**IPL Identification**	**Dose** **(fx)**	EQD2* of IPL dose (Gy)	**PTV Expansion**	**OAR Constraints**	**Pelvic Lymph Nodes Irradiation**	**Androgen Deprivation** **Therapy**	**Clinical Outcome** **(standard vs focal boost, if present)**	**Acute GU**	**Acute GI**	**Late GU**	**Late GI**	**Quality** **of** **Life**
											**≥ G2 (≥ G3)** **(standard vs focal boost, if present)**				
Onal C et al., 2025 [18]	Retrospective study	total: 417 (285); standard: 209 (135); focal boost: 208 (150)	mpMRI	Prostate + SVs: 78 Gy IPL: 86 Gy (39 fx)	91	Prostate + SVs: 8 mm (5 mm posterior) IPL: 4 mm	Rectum V65Gy < 20%, V50Gy < 50% Bladder V65Gy < 30%, V55Gy < 50%	HR, 46–54 Gy (elective lymph nodes)	82.8% (standard), 77.5% (focal boost);6 Mos (IR) or 24–36 Mos (HR)	8-year bDFS: 83.5% vs 93.8%, p = 0.006; 8-year local/regional FFS: 93.1% vs 98.2%, p = 0.03; 8-year distant FFS: 87% vs 93.9%, p = 0.003; 8-year PCSS: 92.3% vs 95.7%, p = NS	23.9% vs 14.9%, p = 0.03	15.3% vs 9.6%, p=NS	10.5% vs. 10.1%, p = NS	6.5% vs 7.8%, p = NS	NR
Onal et al., 2023 [19]	Retrospective study	619 (223)	mpMRI	Prostate + SVs: 78 Gy; IPL: 86 Gy (39 fx)	91	Prostate + SVs: 8 mm (5 mm posterior) IPL: 4 mm	NR	HR, 46–54 Gy (elective lymph nodes)	55.4%;starting before or concurrently; 6–12 Mos (IR), or 24–36 Mos (HR)	5-year bDFS: 93.2%; 5-year PFS: 83.2%; 5-year PCSS: 98.6%	14.6% (0.2%)	7.1% (0%)	6.8% (1.5%)	5.7% (1.1%)	NR
Kuisma et al., 2022 [20]	Prospective study	30 (8)	C-11 acetate PET/CT	Prostate: 76.6 Gy; SVs: 72.9 Gy; IPL: 80.4 Gy (38 fx)	83.1	Prostate: 8–10 mm IPL: 0–6 mm	NR	No	NR	10-year bDFS: 68%; 10-year PCSS: 86%;	[at 3, 12-month visit] ≤ G2: 7%	[at 3, 12-month visit] ≤ G2: 17%	[at 5-year visit]17% (0%)	[at 5-year visit] 4% (4%)	NR
NCT03030625 Zapatero et al., 2022 [22]	Prospective study	30 (11)	mpMRI	Prostate + SVs: 75.95 Gy; IPL: 85.05 Gy (35 fx)	95.5	Prostate + SVs: 7–9 mm (5–7 mm posterior) IPL: 3 mm (2 mm posterior)	Rectum V72Gy < 21%, V58Gy < 40%, Dmean < 46 Gy, Dmax < 79 Gy Bladder V67Gy < 40%, Dmax < 80 Gy	No	50%; 6 Mos (unfavorable-IR) or 18–28 Mos (HR)	30-Month bDFS: 100%; Complete mpMRI response***: 100%	20% (0%)	0%	0%	0%	GU: NS; GI: deteriorated after RT, back to baseline by month 6; sexual function: declined, back to baseline by month 12
NCT01411319 Pollack A et al., 2020 [25]	Prospective phase I trial	25 (4)	mpMRI	Prostate + SVs: 76 Gy (38 fx) IPL: 76 Gy (38 fx) + 12–14 Gy (1 fraction)	122.3–138	Prostate + SVs: 3–5 mm IPL: up to 6 mm (above and below)	Anorectum V65Gy ≤ 17%, V40Gy ≤ 35%; for 12–14 Gy/fx: V3 < 8%, Dmax < 4 Gy Bladder V65Gy ≤ 25%, V40Gy ≤ 50%; for 12–14 Gy/fx: V3Gy < 8%, Dmax < 4.0 Gy Urethra for 12–14 Gy/fx: V3Gy < 10%, Dmax < 6 Gy	HR, 56 Gy (in 38 fx)	56%;4–6 Mos	66-Month bDFS: 92%	52% (0%)	20%	20% (4%)	16%	NR
FLAME (NCT01168479.) Monninkhof et al., 2018 [27], Kerkmeijer et al., 2021 [24], Groen et al., 2022[ [23]	Phase III RCT	total: 571 (479); standard: 287 (240); focal boost: 284 (239)	mpMRI	Prostate + SVs: 77 Gy; IPL: 95 Gy (35 fx)	114.4	Prostate: 5–8 mm IPL: 0 mm	** Rectum V77Gy < 1 cm^3^ ; V50Gy < 50%, V72Gy < 5% or V42.9 Gy < 50%; V66.7 Gy < 25% or Rectal wall V64Gy < 35%, PRV 2 mm: V80Gy < 1 cm^3^ or Rectal Wall PRV 2 mm: Dmean < 30 Gy, V50Gy < 50%, V65Gy < 40%; V70Gy < 30%; V75Gy < 10% Bladder V80Gy < 1 cm^3^ ; V72Gy < 10% or V66.7 Gy < 50%, V71.4 Gy < 25% or no constraints	No	66% (standard), 66% (focal boost);6–36 Mos	5-year bDFS: 85% vs 92%, p < 0.001; 6-year local FFS HR: 0.33, p = 0.01; 6-year regional/distant FFS HR: 0.56, p = 0.02	46% vs 42.3%, p=NS	10.1% vs 14.8%, p=NS	23% (3.5%) vs 27.8% (5.6%), p=NS	12.2% (1.4%) vs 12.7% (1.4%), p=NS	standard vs focal boost: NS
Kao J et al., 2017 [29] ****	Retrospective study	total: 50 (19); standard: 28; focal boost: 22	mpMRI	Prostate: 75.6–79.2 Gy Proximal SVs: 70 Gy Distal SVs: 46.8–57.6 Gy IPL: 79.2–81 Gy (42–44 fx)	71.3–79.4	Prostate: 5–8 mm (3–5 mm posterior)IPL: 3 mm	Rectum Dmax (0.1 cm^3^ ) ≤ Rx dose*****, V75Gy < 15%, V70Gy < 25%, V65Gy < 35%, V50Gy < 50% Bladder V75Gy < 25%, V70Gy < 35%, V65Gy < 50%	45–46.8 Gy (very HR) or 54–59.4 Gy (grossly positive)	54%;6 Mos (IR) or 24 Mos (HR)	2-year bDFS: 98%; 2-year OS: 93%	56%	16%	5%	3% (0%)	GU: NS; sexual function: deteriorated
Garibaldi E et al., 2016 [32]	Retrospective Study	15 (1)	mpMRI	Prostate: 75.2 Gy SVs: 67.2 Gy IPL: 83.2 Gy (32 fx)	97.5	Prostate: 7 mm (5 mm posterior) SVs: 7 mm (5 mm posterior) IPL: 6 mm	[Actual] Rectum V50Gy ≤ 32.5%, V70Gy ≤ 9.7%, V75Gy ≤ 1.2%, D1 ≤ 74.4 Gy Bladder V60GGy ≤ 17.5%, D1 ≤ 77.1GyUrethra D1% ≤ 79.3 Gy	No	80%;6 Mos	16-Month bDFS: 100%	13.3% (0%)	6.6% (0%)	0%	0%	NR
Uzan et al., 2016 [31]	Prospective study	11 (11)	mpMRI	Prostate: 75.4 Gy; IPL: 86 Gy (37 fx)	94	Prostate + SVs: 5–9 mm (2 mm posterior) IPL: 5 mm	[Actual] Rectum V40Gy < 44.7%, V65Gy < 9.9%, V70Gy < 1.2%, Dmax < 74 Gy; Bladder V40Gy < 21.4%, V65Gy < 6.2%, V70Gy < 2.9%, V74Gy < 0.6%;Urethra Dmax < 74.5 Gy	No	starting before radiotherapy;6 Mos or 3 Yrs	3-year bDFS: 90.9%	≤ G2 36.4%	9.1% (0%)	18.2% (0%)	9.1% (0%)	NR
Schild et al., 2014 [34] Laughlin et al., 2022 [21]	Retrospective study	78 (17)	mpMRI	Prostate: 77.4 Gy; Involved SVs: 75–77.4 Gy; Uninvolved SVs:54 Gy; IPL: 81–83 Gy (43 fx)	78.3–81.3	Prostate: 3 mm IPL: 0 mm	Rectum V70Gy ≤ 30%; V75Gy ≤ 10%, V81Gy ≤ 1.8 cm^3^ ; Bladder V70Gy ≤ 30%; V75Gy ≤ 10%, V81Gy ≤ 1.8 cm^3^	No	41%;6 Mos (IR), or 24–36 Mos (HR)	10-year bDFS: 77%; 10-year local FFS: 99%; 10-year distant FFS: 88%; 10-year OS: 66%	54% (0%)	21% (0%)	29% (3%)	4% (0%)	NR
Pinkawa et al., 2012 [35] Schlenter et al., 2018 [28]	Retrospective study	total: 134 (50); standard: 67 (31); focal boost: 67 (19)	18F-choline PET/CT	Prostate + SVs: 76 Gy; IPL: 80 Gy (38 fx)	82.4	Prostate + SVs: 8 mm lateral/anterior, 5 mm superior/inferior, 4 mm posterior IPL: 4 mm (3 mm posterior)	Rectum V70Gy ≤ 20%, V50Gy ≤ 50%, Dmax ≤ 76 Gy Bladder V70Gy ≤ 30%, V55Gy ≤ 50%	No	22% (standard), 13% (focal boost); starting before radiotherapy	5-year bDFS: 85% vs 92%, p=NS; 5-year distant FFS: 100% vs 100%; 5-year PCSS: 100% vs 100%; 5-year OS: 88% vs 100%, p = 0.06	NR	NR	NR	NR	[standard vs focal boost] GU: NS GI: bother scores decrease 2 vs 5; function problems 2% vs 15%, p = 0.03
Ippolito et al., 2012 [36] Buwenge et al., 2020 [26]	Prospective study	44 (20)	T2W-MRI	Prostate + SVs: 72 Gy; IPL: 80 Gy (40 fx)	80	Prostate + SVs: 10 mm (8 mm posterior) IPL: 15 mm (13 mm posterior)	Rectum V65Gy < 30%, V70Gy < 15%, V75Gy < 3% Bladder V50Gy < 50%, V60Gy < 25%, V70Gy < 5%	No	100% starting before radiotherapy;6 Mos (low or IR) or 24 Mos (HR)	10-year bDFS: 90.1%; 10-year local FFS: 94.9%; 10-year regional/distant FFS: 97.6%; 10-year OS: 87.8%	32.5% (2.5%)	20% (5%)	[at 5 years]9.1% (6.8%)	[at 5 years]13.8% (2.3%)	NR
Wong et al., 2011 [37] Schild et al., 2017 [30]	Prospective study	71 (10)	Indium-111-capromab pendetide (ProstaScint) imaging (SPECT)	Prostate + involved SVs: 75.6 Gy; Uninvolved SVs: 50.4 Gy IPL: 82 Gy (42 fx)	80.9	Prostate + SVs: 6 mm IPL: 0 mm	Rectum V65Gy ≤ 40%, V70Gy ≤ 30%, V75Gy ≤ 10%, V81Gy ≤ 1.8 cm^3^ Gy ≤ 30%, V75Gy ≤ 10%, V81Gy ≤ 1.8 cm^3^	No	24%; starting in the last week of radiotherapy;6 Mos (IR) or 12 Mos (HR)	10-year bDFS: 85%; 10-year local FFS: 95%; 10-year regional/distant FFS: 91%; 10-year OS: 69%	55.4% (1.4%)	45% (0%)	44.6% (5.6%)	21% (0%)	NR
Fonteyne et al., 2008 [38] Sundahl et al., 2016 [33]	Retrospective study	total: 410 (197); standard: 185 (74); focal boost: 225 (123)	mpMRI	Prostate + SVs: 78 Gy; IPL: 82 Gy (38 fx)	85.7	Prostate + SVs: 7 mm IPL: 0 mm	Rectum V70Gy ≤ 30%, V65Gy ≤ 40%, V60Gy ≤ 50%, V50Gy ≤ 100%, Dmax ≤ 76 Gy Bladder Dmax ≤ 80 Gy	No irritation;Pelvic lymph node dissection	6 Mos (IR), or 2–3 Yrs (HR)	6-year bDFS: 85%±3% vs 84%±3%, p=NS	51% (5%) vs 45% (7%), p=NS	10% (0%) vs 10% (0%), p=NS	33% (8%) vs 29% (5%), p=NS	12% (2%) vs 8% (0%), p=NS	NR

Footnotes:

* EQD2 calculated using α/β = 1.5.

** Dose constraints might be different among medical centers.

*** “Complete mpMRI response” was defined as “complete disappearance of IPL”.

**** Only the group using focal boost radiotherapy is listed in the table.

***** Rx dose: prescription dose.

fx, fractions(s).

NR, not reported.

NS, not significant.

IR, intermediate risk.

HR, high risk.

Mos, months.

Yrs, years.

bDFS, biochemical disease-free survival.

FFS, failure-free survival.

PFS, progression (including death)-free survival.

Regional PFS: defined as relapse confined to regional lymph nodes.

OS, overall survival.

PCSS, prostate cancer-specific survival.

**Table 2:** Overview of moderate-hypofractionation focal boost EBRT regimens.

**Study** **Author** **Year**	**Study Type**	**Sample** **Size (HR)**	**IPL Identification**	**Dose**	EQD2* of IPL dose (Gy)	**PTV Expansion**	**OAR Constraints**	**Pelvic Lymph Nodes Irradiation**	**Androgen** **Deprivation** **Therapy**	**Clinical Outcome** **(standard vs focal boost, if present)**	**Acute GU**	**Acute GI**	**Late GU**	**Late GI**	**Quality of Life**
											**≥G2 (≥ G3)** **(standard vs focal boost, if present)**				
UMIN000033344 Aizawa et al., 2025 [39]	Prospective study	26 (22)	mpMRI	Prostate + SVs: 54 Gy IPL: 57 Gy (15 fx, in 3 weeks)	86.3	Prostate + SVs: 8 mm (6 mm posterior) IPL: 3 mm	Rectum wall V30Gy ≤ 60%, V45Gy ≤ 30%, V50Gy ≤ 25%, V57Gy ≤ 1% Bladder wall V30Gy ≤ 60%, V50Gy ≤ 30%	No	100%; starting before radiotherapy;6–12 Mos	5-year bDFS: 75.2%; 5-year cFFS: 80.5%	26.9% (0%)	7.7% (0%)	15.4% (0%)	3.8% (0%)	NR
BIOPROP20 (NCT02125175) Chatterjee et al., 2024 [40]	Prospective phase II trial	61 (45)	mpMRI + 18F-choline-PET/CT	Prostate + proximal SVs: 60 Gy Distal SVs: 47 Gy IPL: 68 Gy Lymph nodes: 45–50 Gy (20 fx)	95.2	Prostate + SVs: NRIPL: 3 mm	Rectum V24.6 Gy < 70%, V32.4 Gy < 60%, V40.8 Gy < 50%, V48.6 Gy < 35%, V52.8 Gy < 30%, V57Gy < 15%, V60Gy < 3%, V64Gy < 0%, Dmean ≤ 35 Gy Bladder V40.8 Gy < 50%, V48.6 Gy < 25%, V60Gy < 5% Urethra D2% ≤ 61 Gy	8.2%; elective nodes: 45 Gy; involved nodes: 50 Gy	100%; starting before radiotherapy;6 Mos, or 2–3 Yrs	5-year cFFS: 88.5%; 5-year MFS: 82.4%; 5-year OS: 91.2%; 5-year PCSS: 98.1%	crude: 22.9% (1.6%); cumulative: 49.2%	crude: 4.9% (0%);cumulative: 6.6% (0%)	30.1% (0%)	3.3% (0%)	GU: worst at week 6, back to baseline at week 18
Hypo-Focal (ARO2020-01) Zamboglou et al., 2022 [43]	Prospective phase II trial	25 (6) **	mpMRI + PET/CT (^68^Ga-PSMA/^18^F-PSMA)	Prostate: 60 Gy IPL: up to 75 Gy (median mean dose 70 Gy, range 64–75 Gy) (20 fx)	112.5 (median mean dose 100, 85.9–112.5)	Prostate + SVs: 4–9 mm IPL: 2–4 mm	Rectum V25Gy < 80%, V32Gy < 65%, V49Gy < 35%, V53Gy < 30%, V57Gy < 15%, V60Gy < 3%, V64Gy < 1 cm^3^ Bladder V41Gy < 50%, V49Gy < 25%, V60Gy < 5%, V67Gy < 1 cm^3^	No	38%; 6 Mos (IR) or 12 Mos (HR)	Before- vs after- radiotherapy: IPL-MRI: 1.4 cm^3^ vs 0.6cm^3^ADCmean: 931 vs 1157 × 10^−6^ mm^2^/s	[6 months]36% (0%)	[6 months]16% (0%)	NR	8% (8%)	GU: worst at week 4, back to baseline by month 2
DELINEATE (ISRCTN 04483921) Murray et al., 2020 [46], Tree et al., 2023 [42]	Prospective phase II trial	total: 256 (145); arm A: 55 (15); arm B: 153 (82);arm C: 48 (48)	mpMRI	arm A: Prostate + SVs: 74 Gy IPL: 82 Gy (37 fx, in 7.5 weeks) arm B: Prostate + SVs: 60 Gy IPL: 67 Gy (20 fx, in 4 weeks) arm C: Prostate + SVs: 74 Gy IPL: 82 Gy Lymph Nodes: 60 Gy (37 fx, in 7.5 weeks)	arm A/C: 87.1arm B: 92.8	Prostate + SVs: 3–6 mm (0–6 mm posterior) IPL: 2 mm (excluding the urethra) Lymph nodes: 5 mm	arm A/C Rectum V30Gy < 80%, V40Gy < 65%, V50Gy < 50%, V60Gy < 35%, V65Gy < 30%, V70Gy < 15%, V75Gy < 3%, D2% < 76 Gy Bladder V50Gy < 50%, V60Gy < 25%, V65Gy < 50%, V70Gy < 5%, V75Gy < 3%, V80Gy < 0.2% Urethra D2% < 77 Gy arm B Rectum V24.3 Gy < 80%, V32.4 Gy < 65%, V40.5 Gy < 50%, V48.7 Gy < 35%, V52.7 Gy < 30%, V56.8 Gy < 15%, V60.8 Gy < 3%, D2% < 61.6 Gy Bladder V40.5 Gy < 50%, V48.7 Gy < 25%, V52.7 Gy < 50%, V56.8 Gy < 5%, V60.8 Gy < 3%, V64.9 Gy < 0.2% Urethra D2% < 62.4 Gy	arm C, 60 Gy (37 fx)	100%; starting before radiotherapy;6 Mos or 2–3 Yrs	arm A vs arm B vs arm C: 5-year b/c FFS: 98.2% vs 96.7% vs 95.1%	arm A vs arm B vs arm C: [RTOG]38.2% vs 35.9% vs 37.5%	arm A vs arm B vs arm C: [RTOG] 10.9% vs 13.7% vs 14.6%	arm A vs arm B vs arm C: [RTOG] 12.9% (3.7%) vs 18.2% (2%) vs 18.2% (2.5%) [CTCAE] 21.5% (1.9%) vs 27.2% (0%) vs 20.7% (2.5%)	arm A vs arm B vs arm C: [RTOG] 12.8% (0%) vs 14.6% (0.9%) vs 20.7% (2.7%) [CTCAE] 18.8% (0%) vs 17.8% (0.7%) vs 18.1% (5.4%)	GU: NS; GI: worst at yr 1, back to baseline by yr 2; sexual domain: back to baseline by yr 2
Kao J et al., 2020 [45]	Retrospective study	total: 94 (25) (focal boost 77); conventional: 85;moderate: 9	mpMRI	conventional: Prostate + proximal SVs:76–79.2 Gy Distal SVs: 50.4–54 Gy IPL: 80–81 Gy (42-44fx) moderate: Prostate + proximal SVs: 60 Gy Distal SVs: 50.4–54 Gy IPL: 62 Gy (20 fx)	conventional: 75.8–79.4moderate: 81.5	Prostate + SVs: 5–8 mm (3–5 mm posterior) IPL: 3 mm	conventional: Rectum Dmax (0.1 cm^3^) ≤ Rx dose***, V75Gy < 15%, V70Gy < 25%, V65Gy < 35%, V50Gy < 50% Bladder V75Gy < 25%, V70Gy < 35%, V65Gy < 50%moderate: NR	16%; elective nodes: 45 Gy; involved nodes: 54–59.4 Gy	81%; 6 Mos (high IR) or 18–36 Mos (high to very HR)	3-year bDFS: 96%	54% (0%)	12% (0%)	7% (0%)	3% (0%)	NR
NCT01921803 Fonteyne et al., 2018 [47], 2024 [41]	Prospective phase III RCT	total: 342 (181); arm A: 170 (90); arm B: 172 (91);(focal boost 79% in each arm)	bpMRI	arm A: Prostate + involved SVs: 56 Gy Uninvolved SVs: 50 Gy IPL: D98 = 57 Gy (16 fx, 4 fx/week) arm B: Prostate + involved SVs: 67 Gy Uninvolved SVs: 50 Gy IPL: D98 = 68 Gy (25 fx, 5 fx/week)	arm A: 82.5arm B: 82	Prostate + SVs: 5 mm IPL: 0 mm	arm A: Rectum/Sigmoid DmaxGy < 57.6 Gy, V33Gy < 46%, V40Gy < 34%, V47Gy < 25%, V51Gy < 20%, Dmean < 30 Gy Bladder Dmax < 59.5 Gy Urethra Dmax < 59.5 Gy arm B: Rectum/Sigmoid Dmax < 66.9 Gy, V33Gy < 46%, V46Gy < 34%, V54Gy < 25%, V59Gy < 20%, Dmean < 33 Gy Bladder Dmax < 69.3 Gy Urethra Dmax < 69.3 Gy	No	86%; ≤ 6 Mos (44%) or > 6 Mos (42%)	NR	arm A vs arm B: 61% (9%) vs 60% (7%)	arm A vs arm B:24% (1%) vs 24% (0%)	arm A vs arm B:47% (10%) vs 42% (6%)	arm A vs arm B:22% (3%) vs 19% (4%)	NR
COA no. Si 691/2018 Dankulchai et al., 2022 [44]	Prospective study	total: 45 (42); moderate: 13; conventional: 32	mpMRI	moderate: Prostate + involved SVs: 60 Gy Uninvolved SVs: 50 Gy IPL: 70 Gy (20 fx) conventional: Prostate + involved SVs: 78 Gy Uninvolved SVs: 60 Gy IPL: 87.75 Gy (39 fx)	moderate: 100 conventional: 94	Prostate + SVs: 5 mm (3 mm posterior) IPL: 5 mm (3 mm posterior)	moderate Rectum V60Gy < 5%, V57Gy < 15%, V37Gy < 50% Bladder V60Gy < 5%, V48.6 Gy < 25%, V40.8 Gy < 50% conventional Rectum V65Gy < 17%, V40Gy < 35% Bladder V65Gy < 25%, V40Gy < 50%	No	100%;	NR	moderate vs conventional: 30.7%, vs 28.1%, p=NS	moderate vs conventional: 15.3% vs 6.3%, p=NS	Total: crude: 8.9% (0%); incidence: 12.6% (0%)	Total: crude: 2.2% (0%); incidence: 2.8% (0%)	GU: deteriorated after radiotherapy, back to baseline at month 3
Onjukka et al., 2017 [48]	Prospective study	28 (28)	mpMRI	Prostate: 60 Gy SVs: 53 Gy IPL: 68 Gy (20 fx)	95.2	Prostate + SVs: 5–9 mm IPL: 5 mm	PRV: 2 mm added to rectum, bladder and urethra Rectum V24.6 Gy < 70%, V32.4 Gy < 60%, V40.8 Gy < 50%, V48.6 Gy < 35%, V52.8 Gy < 30%, V57Gy < 15%, V60Gy < 3%, V64Gy < 0%, V68Gy < 0%, D2% ≤ 58 Gy, Dmean ≤ 35 Gy Bladder V40.8 Gy < 50%, V48.6 Gy < 25%, V60Gy < 5% Urethra D2% ≤ 61 Gy	No	100%; starting before radiotherapy;6 Mos or 2–3 Yrs	37-month b/cFFS: 89.3%	NR	NR	7.1% (0%)	0%	NR
Project EC UZG 2006/018 Fonteyne et al., 2013 [49]	Prospective study	total: 80 (80); standard: 20;focal boost: 60	MRI+/- MRS	Prostate + SVs: 69.3 Gy (median) IPL: 72 Gy (median) Lymph Nodes: 45–65 Gy (25 fx)	90.1	Prostate + SVs: 7 mm IPL: 0 mm Lymph nodes: 7 mm	Rectum D30% < 63.3 Gy, D40% < 60.1 Gy, D50% < 56.9 Gy or V36Gy < 84%, V45Gy < 69%, V54Gy < 59%, V57Gy < 48%, V66Gy < 30%, Dmax < 66.9 Gy Bladder Dmax < 69.3 Gy	100%, regional lymph node (lnn) chains: ≥45 Gy (25 fx); pathologically enlarged lymph nodes: 65 Gy (25 fx)	100%; starting before radiotherapy;2–3 Yrs	3-year bDFS: 81%; 3-year cFFS: 89%; 3-year PCSS: 95%; 3-year OS: 88%	63.8% (7.5%)	58.8% (1.3%)	crude: 34% (5%);actuarial: 40% (6%)	crude: 23% (6%);actuarial: 28% (8%)	NR

Footnotes.

*EQD2 calculated using α/β = 1.5.

**Only the group using external beam radiation is listed in this table.

fx, fraction(s).

NR, not reported.

NS, not significant.

IR, intermediate risk.

HR, high risk.

Mos, months.

Yrs, years.

bDFS, biochemical disease-free survival.

FFS, failure-free survival.

cFFS, clinical failure-free survival.

PFS, progression (including death)-free survival.

Regional PFS was defined as relapse confined to the regional lymph nodes.

MFS, metastasis-free survival.

OS, overall survival.

PCSS, prostate cancer-specific survival.

**Table 3:** Overview of ultra hypofractionation focal boost EBRT regimens.

**Study** **Author,** **Year**	**Study Type**	**Sample Size(high risk)**	**IPL Identification**	**Dose**	EQD2* of IPL dose (Gy)	**PTV Expansion**	**OAR Constraints**	**Pelvic Lymph Node Irradiation**	**Androgen** **Deprivation** **Therapy**	**Clinical Outcome** **(standard vs focal boost, if present)**	**Acute GU**	**Acute GI**	**Late GU**	**Late GI**	**Quality of Life**
											**≥G2 (≥ G3)** **(standard vs focal boost, if present)**				
PROBE Singh et al., 2025 [50]	Prospective phase II trial	30 (14)	mpMRI + 68 Ga-PSMA-PET/CT	Prostate: 36.25 Gy IPL (PET ∪ MRI): 40 Gy IPL (PET ∩ MRI): 42.5 Gy Lymph nodes: 25 Gy (5 fx, every other day)	108.6–121.4	Prostate: 5 mm IPL: 0 mm lymph nodes: 5 mm	Rectum V14Gy < 45%, V17.5 Gy < 30%, V28Gy < 12%, V31.5 Gy < 8%, V35Gy < 3% Bladder V14Gy < 30%, V17.5 Gy < 25%, V28Gy < 10%, V31.5 Gy < 5%, V35Gy < 3% Urethra Dmax 0.1 cm^3^ < 40 Gy, Dmean < 90% of DIL Rx	100%, 25 Gy (5 fx)	100%; starting concurrently; 6 Mos	NR	13.3% (0%)	6.6 (0%)	NR	NR	NR
Tsurugai Y et al., 2024 [59]	Retrospective study	520 (230)	mpMRI	Prostate: 35–36.25 Gy IPL: 115–140% of Prostate (5 fx, 1–3 fx/week)	109.8–168.9	Prostate + SVs: 3 mm (6 mm around IPL) IPL: 3 mm	PRV: 3 mm margin added to rectum, bladder and urethra; Rectum D0.035 cm^3^ ≤ 100% Rx dose; PRV ≤ 105% Rx dose; Bladder D0.035 cm^3^ ≤ 100% Rx dose; PRV ≤ 105% Rx dose; Urethra D99% ≥ 100% Rx dose; PRV ≤ 105% Rx dose	No	65%	4-year bDFS: 94.8%; 4-year OS: 95.7%	22.3% (0%)	2.1% (0%)	7.5% (0%)	0.8% (0%)	NR
SMILE Fink et al., 2024 [55]	Multicenter prospective phase II trial	total: 69 (3); standard: 52; focal boost: 17	mpMRI	Prostate + SVs: 37.5 Gy IPL: 40 Gy (5 fx, every other day)	108.6	Prostate + SVs: 3 mm IPL: 0 mm	PRV: 2 mm margin added to urethra; Urethra D0.2 cm^3^ ≤ 37.5 Gy	No	12%;starting before radiotherapy; ≤ 3 Mos	NR	[RTOG]15% (1%) [CTCAE] 17% (0%)	[RTOG] 21% (4%) [CTCAE] 9% (0%)	NR	NR	GU, GI: symptoms subsided after 6–12 weeks; emotional score: improved significantly
DESTROY-4 Deodato et al., 2024 [58]	Prospective phase I trial	24 (0)	mpMRI	Prostate + SVs: 35 Gy IPL: 40–50 Gy (5 fx, once a day)	108.6–164.3	Prostate + SVs: 3 mm IPL: 3 mm	Rectum D0.035 cm^3^ < 38 Gy; Bladder D0.035 cm^3^ < 42 Gy; Urethra D0.035 cm^3^ < 44 Gy	No	66.7%； starting 3 Mos before radiotherapy;6 Mos	2-year bDFS: 100%	12.5% (0%)	12.5% (0%)	4.2% (0%)	12.5% (0%)	GU: worst at month 1; recovery to baseline at yr 1
HERMES (NCT04595019) Westley et al., 2024 [57]	Prospective phase II trial	total: 20 (5); standard: 10 (2); focal boost: 10 (3)	mpMRI	standard Prostate: 40 Gy SVs: 30–36.25 Gy (5 fx, in 2 weeks) focal boost Prostate + SVs: 24 Gy IPL: 27 Gy (2 fx, in 8 days)	115.7	Prostate + SVs: 3 mm IPL: 0 mm	Rectum V20.8 Gy < 1 cm^3^, V17.6 Gy < 4 cm^3^, V13Gy < 7 cm^3^ Bladder V20.8 Gy < 5 cm^3^, V14.6 Gy < 15cm^3^Urethra D10% < 26 Gy	No	100%; starting before radiotherapy;3–12 Mos	NR	10% (0%) vs 20% (0%)	0% vs 0%	NR	NR	NR
SABR-Dual (NCT06027892) Fredman et al., 2024 [56]	Prospective phase I trial	total: 20 (0); standard: 10; focal boost: 10	mpMRI	*** Prostate + proximal SVs: 27 Gy Distal SVs: 23 Gy IPL: 30 Gy (2 fx, 3–7 days between fx)	141.4	Prostate + SVs: 2 mm IPL: 0 mm	PRV: 2 mm margin added to urethra; Rectum D0.03 cm^3^ < 27 Gy, D1cm^3^ < 22.95 Gy, V23 ≤ 10%, V19 ≤ 20%, V10 ≤ 50%; Rectal Wall 35% circumference < 20 Gy, 50% circumference < 13.5 Gy; Bladder D0.03 cm^3^ < 28.08 Gy, V16Gy ≤ 25%, V16.2 Gy ≤ 50 cm^3^; Urethra D0.03 cm^3^ ≤ 27 Gy; PRV D0.03 cm^3^ < 27.54 Gy	No	No	NR	[≤180 days from the start of radiation]15% (0%)	0%	NR	NR	Highest urinary incontinence MCIC: 15%; Highest urinary obstructive MCIC: 30%; Highest bowel MCIC: 10%
Hypo-FLAME 2.0 (NCT04045717) De Cock et al., 2023 [65]	Prospective phase II trial	124 (83)	mpMRI	Prostate: 35 Gy SVs: 30 Gy IPL: 50 Gy (5 fx, twice a week)	164.3 (actual median mean dose: 146.4)	Prostate + SVs: 4–5 mm IPL: 0 mm	PRV: 2 mm margin added to rectum and urethra; Rectum V38Gy < 1 cm^3^, V35Gy < 2 cm^3^, V32Gy < 15%, V28Gy < 20%, Dmax (0.035 cm^3^) < 40 Gy; Bladder V42Gy < 1 cm^3^, V37 < 5 cm^3^, V32Gy < 15%, V28Gy < 20%; Urethra Dmax (0.035 cm^3^) < 42 Gy;	No	62.1%;starting concomitantly	NR	47.5% (0%)	7.4% (0%)	NR	NR	Acute GU MCIC: 74.1%;Acute GI MCIC: 47.4%
NCT02470897 Morris et al., 2023 [61]	Prospective phase II trial	total: 114 (0); standard: 14; focal boost: 100	mpMRI	standard: Prostate + SVs: 37.5 Gy (5 fx, every other day) focal boost: Prostate + SVs: 40 Gy IPL: 42.5–45 Gy (40 Gy within PRV) (5 fx, every other day)	121.4–135	Prostate + SVs: 4 mm (3–4 mm anterior, 1.5–2.5 mm posterior) focal boost: Prostate + SVs: 3–4 mm (1.5–2.5 mm posterior) IPL: 0 mm	PRV: 2 mm margin added to anterior rectal wall, bladder base and urethra; Rectum V42Gy < 0.03 cm^3^, V38Gy < 3 cm^3^, V36Gy < 10%, V32Gy < 20%, V18Gy < 50%; PRV Dmax < 36.25 Gy; Bladder V42Gy < 0.03 cm^3^, V36Gy < 10%, V20Gy < 50%; PRV Dmax < 36.25 Gy;Urethra PRV Dmax < 36.25 Gy	No	35% of focal boost	5-year bDFS: 100% vs 97% 5-year OS: 96.5%	[6 weeks cumulative]36% (0%) vs 38% (0%)	[6 weeks cumulative]0% vs 4% (0%)	7% vs 14%	0% vs 6% (0%)	GU, sexual function: NS; GI: worst at month 12, back to baseline later
SPORT (NCT03253978) Houlihan et al., 2023 [63]	Prospective Phase III RCT	total: 30 (25); standard: 21; focal boost: 9	mpMRI	PTVprostate + proximal SVs: 36.25 Gy CTVprostate + proximal SVs: 40 Gy Distal SVs: 25 Gy IPL: 45–50 Gy (5 fx, once a week)	135–164.3	Prostate + SVs: 5 mm IPL: 3 mm Lymph nodes: 5–7 mm	PRV: 3 mm margin added to urethra; Rectum V18.1 Gy < 50%, V29Gy < 20%, V36Gy < 1 cm^3^ Bladder V18.1 Gy < 40%, V37Gy < 10 cm^3^ Urethra V42Gy < 50%, V45Gy < 0.01 cm^3^; PRV V42Gy < 50%, V45Gy < 0.01 cm^3^	50%, 25 Gy in 5 fx	100%;starting before radiotherapy; 12–36 Mos	NR	****14.3% (0%) vs 11.1% (0%)	4.8% (0%) vs 0%	28.6% (4.8%) vs 0%	14.3% (0%) vs 0%	Acute GU MCIC: 62% vs 44%; Acute GI MCIC: 38% vs 44%; Late GU MCIC: 76% vs 67%; Late GI MCIC: 52% vs 33%
2SMART (NCT03588819) Ong et al., 2023 [62]	Prospective phase II trial	30 (0)	mpMRI	Prostate + SVs: 26 Gy IPL: 32 Gy (2 fx, once a week)	160	Prostate + SVs: 2.5 mm (2 mm superior/inferior) IPL: 0 mm	** Rectum V23Gy < 0.67 cm^3^, V22Gy < 0.99 cm^3^; Bladder V22Gy < 3.17 cm^3^, V20.8 Gy < 6.73 cm^3^; Urethra PRV Dmax < 33.62 Gy, V32.5 Gy < 0.01 cm^3^	No	7%	44-month bDFS: 92%	56.67% (0%)	3.33% (0%)	50% (0%)	10% (0%)	Acute GU MCIC: 33%; Acute GI MCIC: 20%; Late GU MCIC: 50%; Late GI MCIC: 30%
Brennan et al., 2023 [64]	Retrospective study	15 (0)	mpMRI	Prostate + SVs: 40 Gy IPL: 45 Gy (5 fx, every other day)	135	Prostate + SVs: 3 mm IPL: 2 mm	Rectum D0.035 cm^3^ ≤ 41.2 Gy, D1cm^3^ < 38.5 Gy, Mean Dose 13 Gy (Guideline)/16.4 Gy (Limit), V24Gy < 25%, V30Gy < 8 cm^3^, V10Gy < 52% (guideline) Bladder D0.035 cm^3^ ≤ 42 Gy, D50% < 20 Gy, D10% < 36 Gy Urethra D0.035 cm^3^ ≤ 42 Gy, D1cm^3^ < 40 Gy	No	No	NR	NR	NR	20%	13.3%	NR
NCT02353819 Hannan et al., 2022 [67]	Prospective phase I trial	55 (55)	mpMRI	Prostate + proximal SVs: 47.5 Gy Distal SVs: 22.5–25 Gy IPL: 50–55 Gy (5 fx; ≤3 times/week, ≥36 h between fx)	164.3–196.4	Prostate + SVs: 3 mm IPL: 0–3 mm Lymph nodes: 5 mm	Rectum V50Gy < 3 cm^3^, V39Gy < 33% circumference, V24Gy < 50% circumference, Dmax (0.03 cm^3^) < 49.875 Gy; Rectum superior to prostate: V25Gy < 10 cm^3^, Dmax (0.03 cm^3^) < 21.375 Gy; Bladder: V18.3 Gy < 50 cm^3^, Dmax (0.03 cm^3^) < 49.875 Gy; Bladder trigone V18.3 Gy < 18 cm^3^; Urethra: Dmax (0.03 cm^3^) < 49.875 Gy	100%, 22.5–25 Gy in 5 fx	100%; starting before radiotherapy;2 years	2-year bDFS: 96.6%; 2-year bPFS: 94.8%; 2-year PCSS: 100%; 2-year OS: 98.2%	25% (0%)	13% (0%)	22% (2%)	7%	Acute GU MCIC: 45%; Acute GI MCIC: 45%
Hypo-FLAME (NCT02853110) Draulans et al., 2020 [75], 2024 [52]	Prospective phase II trial	100 (68)	mpMRI	Prostate + SVs: 35 Gy; IPL: up to 50 Gy (median mean dose 44.7 Gy) (5 fx, once a week)	164.3 (actual median mean dose: 133.3)	Prostate + SVs: 4–5 mm IPL: 0 mm	PRV: 2 mm margin added to rectum and urethra; Rectum V38Gy < 1 cm^3^, V35Gy < 2 cm^3^, V32Gy < 15%, V28Gy < 20%, Dmax (0.035 cm^3^) < 40 Gy; Bladder V42Gy < 1 cm^3^, V37Gy < 5 cm^3^, V32Gy < 15%, V28Gy < 20%; Urethra Dmax (0.035 cm^3^) < 42 Gy	No	62%;≤6 Mos or 6–36 Mos	5-year bDFS: 93% (86%∼97%); 5-year OS: 94% (87%∼97%)	34% (0%)	5% (0%)	28% (2%)	14% (1%)	Acute GU MCIC: 53.6%; Acute GI MCIC: 18.4%; [at 5 years] GU: MCIC 24%; vs baseline: p=NS;GI: MCIC 12%; vs baseline: worse, p = 0.014
5STAR (NCT02911636) Alayed et al., 2020 [68]	Prospective phase II trial	30 (19)	mpMRI	Prostate: 35 Gy IPL: up to 50 Gy (actual D99% 44.6 Gy) Lymph nodes + SVs: 25 Gy (5 fx, once a week)	164.3 (actual D99% 132.8)	Prostate: 2.5 mm (2 mm superior/inferior) IPL: 0 mm Lymph nodes/SVs: 6 mm	Rectum V28Gy < 15%, V35Gy < 5%; Bladder V28Gy < 15%;Urethra Dmax < 52 Gy, D10% < 47.2 Gy	100%, 25 Gy in 5 fx	100%; 6 Mos (unfavorable-IR) or 12–18 Mos (HR)	NR	66.7% (3.3%)	16.67% (0%)	46.67% (0%)	13.33% (0%)	GU MCIC: 34.4%; GI MCIC: 40.7%
SPARC trial (NCT2145494) Nicholls et al., 2020 [69], Yasar et al., 2024 [51]	Prospective phase II trial	20 (16)	mpMRI	Prostate + SVs: 36.25 Gy IPL: 47.5 Gy (5 fx, every other day)	149.3	Prostate + SVs: 5 mm (3 mm posterior) IPL: 0 mm	Rectum V18.1 Gy < 50%, V29Gy < 20%, V36Gy < 1 cm^3^; Bladder V18.1 Gy < 40%, V37Gy < 5 cm^3^;Urethra V42Gy < 50%, V45.6 Gy < 10%	No	100%; starting before radiotherapy;6–18 Mos	30-month bDFS: 100%	25% (5%)	30% (0%)	16.7% (0%)	0%	GU, global status: NS
NCT02254746 Herrera et al., 2019 [71], Cloître et al., 2023 [66], 2024 [53]	Prospective phase I/II trial	33 (18)	mpMRI	Prostate: 36.25 Gy IPL: 45–50 Gy (5 fx; ≤20 days in total, 2–6 days between fx, ≤2 fx/ week)	135–164.3	Prostate: 3 mm IPL: 3 mm	Rectum V25Gy < 20, D1 cm^3^ < 38 Gy, D0.1 cm^3^ < 41 Gy; Bladder Dmedian < 20 Gy, D1 cm^3^ < 38 Gy, D0.1 cm^3^ < 45 Gy; Urethra D1 cm^3^ < 39 Gy, D0.1 cm^3^ < 41 Gy;	No	3%；6 Mos	82-month bDFS: 60.6%; 82-month PCSS: 97%	15% (0%)	6.1% (0%)	21% (3%)	6% (6%)	NR
NCT01856855 McDonald et al., 2019 [72], Maas et al., 2023 [60]	Prospective study	26 (0)	T1W, T2W-MRI	Prostate: 36.25 Gy IPL: 40 Gy (5 fx, in 7–17 days)	108.6	Prostate: 5 mm (3 mm posterior) IPL: 5–10 mm (3–8 mm posterior)	Rectum D1cm^3^ ≤ 38.06 Gy, D3cm^3^ < 34.4 Gy, V36.25 Gy < 5%, V29Gy < 20%, V18.125 Gy < 50%; Bladder D1cm^3^ ≤ 38.06 Gy, V36.625 Gy < 10%, V18.125 Gy < 50%; Urethra Dmax ≤ 38.78 Gy	No	26.9%;6 Mos (IR)	5-year bDFS: 100%; 5-year OS: 100%	7.7% (0%)	NR	38.5% (0%)	11.5% (0%)	GU, GI: worst at month 1, back to baseline later; sexual function: deteriorated
GIVE ME FIVE (AIRC-IG-13218)(NCT01913717) Timon et al., 2018 [73], Marvaso et al.2020 [70], 2024 [54]	Prospective phase II trial	65 (0)	mpMRI	Prostate + SVs: 36.25 Gy IPL: 37.5 Gy (5 fx, every other day)	96.4	Prostate + SVs: 5 mm (3 mm posterior) IPL: 3 mm	Rectum V18Gy < 50%, V29Gy < 29%, V33Gy < 10%, V36Gy < 5%; Posterior rectal wall D1 cm^3^ < 16 Gy; Bladder V36Gy < 10%, V36Gy < 5 cm^3^, V18Gy < 40%;Urethra V36Gy < 50%, Dmax < 40 Gy	No	7.69%	5-year bDFS: 95%; 5-year PFS: 83% (0.74–0.93); 5-year OS: 86%	[at 1 month]7.7% (1.5%)	[after radiotherapy]3.1% (0%)	[after 2 years]4.1% (0%)	[after 2 years]4.1% (0%)	Global status: worst at month 1, back to baseline at yr 1
Aluwini et al., 2013 [74]	Prospective study	total: 50 (0); standard: 36; focal boost: 14	T1W, T2W, DCE-MRI	Prostate: 38 Gy IPL: 44 Gy (4 fx, once a day)	157.1	Prostate: 3 mm IPL: 0 mm	Rectum D1cm^3^ < 32.3 Gy; Anterior rectal wall Dmax < 38 Gy; Rectal mucosa Dmax < 28.5 Gy; Bladder D1 cm^3^ < 38 Gy, Dmax < 41.8 Gy;Urethra D5% < 45.5 Gy, D10% < 42 Gy, D50% < 40 Gy	No	No	2-year bDFS: 100%	23% (8%)	14% (2%)	[at 2 years]16% (6%)	[at 2 years]3%	GU, GI: NS; sexual function: deteriorated

Footnotes:

*EQD2 calculated using α/β = 1.5.

**actual OAR constraints.

***re-calculated based on the reports.

****of the group that has been implemented.

fx, fraction(s).

NR, not reported.

NS, not significant.

IR, intermediate risk.

HR, high risk.

Mos, months.

Yrs, years.

bDFS, biochemical disease-free survival.

FFS, failure-free survival.

PFS, progression (including death)-free survival.

Regional PFS: defined as relapse confined to regional lymph nodes.

OS, overall survival.

PCSS, prostate cancer-specific survival.

The cohorts encompassed prostate cancer patients ranging from those defined by NCCN as very low-risk to very high-risk disease, with the majority comprising high-risk localized prostate cancer, with 51.9% (1415/2725) in the conventional fractionation group, 60.2% (444/737) in the moderate-hypofractionation cohort, and 39% (536/1375) in the ultra hypofractionation cohort. As the only exception, node-positive patients were included in one Phase II trial [49].

Focal boost regimens were derived from standard schemes with adjustments to the fractions and dose. Whole prostate prescriptions were adapted to 1.8–2.35 Gy in 32–45 fractions (conventional fractionation), 2.77–3.6 Gy in 15–25 fractions (moderate hypofractionation), and 7–13.5 Gy in 2–5 fractions (ultra hypofractionation). Assuming an α/β of 1.5 [76], equivalent dose in 2 Gy/fraction (EQD2) to IPLs spanned widely (Fig. S1): most conventional fractionation regimens stayed < 100 Gy (71.3–138 Gy), with the FLAME trial [24] (a randomized phase III trial evaluating focal lesion ablative microboost in prostate cancer) pushing to 114.4 Gy and a spatial fractionation approach [25] reaching 122.3–138 Gy (standard EBRT plus a single 12–14 Gy stereotactic boost). Moderate hypofractionation delivered comparable escalation (81.5–112.5 Gy_1.5_, EQD2 with α/β = 1.5) to conventional cohorts. Ultra hypofractionation delivered higher IPL doses (96.4–196.4 Gy_1.5_, EQD2 with α/β = 1.5) with greater inter-study variability. The most intensely escalated examples were the NCT02353819 (50–55 Gy in 5 fractions; 164.3–196.4 Gy_1.5_, EQD2 with α/β = 1.5) [67] and the 2SMART trial [62] (32 Gy in 2 fractions; 160 Gy_1.5_ EQD2 with α/β = 1.5), yielding the most intense cumulative and per-fraction biological effect, respectively. Delivery frequency ranged from daily to weekly, with the alternate-day schedule and the once-weekly schedule being the two most commonly adopted schedules.

Pelvic lymph node irradiation was of 54–59.5 Gy in three conventional fractionation cohorts, 50–65 Gy in three moderate fractionation cohorts, and 22.5–25 Gy in four ultra hypofractionation cohorts. Three conventional-fractionation and three moderate hypofractionation studies delivered elective pelvic nodal irradiation to high-risk patients concomitantly, at doses of 45–54 Gy. Surgical pelvic lymph node dissection was performed before pelvic irradiation in patients at high risk or with suspicious nodes in one conventional fractionation study [38], and in one moderate hypofractionation study [49].

The delineation of IPLs was based on MRI in 39 of 42 studies, most commonly multiparametric MRI (mpMRI), and on PET/CT in five studies, with overlap between modalities in three studies. One historical report employed ^111^indium capromab pendetide (ProstaScint)-based single-photon emission computed tomography (SPECT) [30]. Among 22 studies that prescribed neoadjuvant hormone therapy, the initial diagnostic MRI was fused to the planning CT/MRI to favor an accurate delineation of IPLs in 15 studies. The volumes of the MRI-defined lesions were 0.67–6.59 cm^3^, and those defined on PET/CT were 2.2–8 cm^3^. Multifocal distribution was observed. Among studies reporting the number of IPLs eligible for focal boost, all visible IPLs were generally boosted, whereas four studies imposed restrictions, targeting only one lesion [50], [58], or up to two [51] or three lesions [42].

In the MRI-based studies, boost targets were typically defined as prostate imaging reporting and data system (PI-RADS) 4–5 lesions. Six studies allowed inclusion of PI-RADS 3 lesions as candidate boost targets [22], [68], particularly when combined with additional imaging modalities such as prostate-specific membrane antigen (PSMA)-targeted PET [43] or with biopsy confirmation [31], [42], [57]. Quantitative imaging parameters were also incorporated to refine contours in one study using an apparent diffusion coefficient (ADC) threshold < 1000 × 10^−6^ mm^2^/s [25]. Delineation of IPLs using PET/CT was performed in five studies, either alone [20], [28] or in combination with mpMRI [40], [43], [50]. Radiotracers utilized included ^11^C-acetate (a radiolabeled acetate tracer reflecting lipid metabolism), ^18^F-choline (a radiolabeled choline analog associated with cell membrane synthesis tracer), and ^68^Ga or ^18^F-labeled PSMA–targeting small-molecule ligands in more recent studies. Delineation criteria varied from absolute standard uptake value (SUV) cutoffs (e.g., a median SUV of 2.9 for ^11^C-acetate [20]) to tumor-to-background uptake ratio > 2 (background SUV defined as the maximum SUV measured in a 1 cm^2^ region within the prostate exhibiting the lowest activity [28]). One trial defined a percentage-of-SUVmax strategy that best matched mpMRI contours (median 48% SUVmax) [50]. Histopathology assisted in IPL targeting by confirming PI-RADS 1–3 foci [31] or indicating suspicious biopsy-positive regions as the boost volume when no discrete IPL was visible on MRI [21].

In conventional fractionation regimens, the planning target volume (PTV) expansion of the prostate was 3–10 mm, with median posterior margins often reduced to 0–8 mm to spare the rectum; PTV expansions of the IPLs were 0–6 mm, except for a margin of 10 mm in one study [26], with four studies applying no additional margin to the IPLs [21], [24], [30], [33] (Fig. S2. A). Moderate hypofractionation regimens used similar margins of 3–9 mm (0–6 mm posteriorly) for the prostate and 0–5 mm for IPLs, with two studies using no IPL margin [41], [49] (Fig. S2. B). Ultra hypofractionation adopted smaller margins: 2–5 mm (1.5–3 mm posteriorly) for prostate and 0–3 mm for most IPLs, with 11 out of 19 studies applying no IPL expansion (Fig. S2. C). Although one study intended margins of 5–10 mm (posterior 3–8 mm) for IPLs, 5 mm (posterior 3 mm) was utilized in 92.3% of patients [60]. The exclusion of the urethra, when overlapping with the extended PTV, was specifically reported in [42].

Organ at Risk (OAR) constraints varied by fractionation schemes and institutions. In conventional fractionation regimens, rectal V70Gy was generally < 30%, with stricter plans setting limits of < 15–25%; when V70Gy was not specified, V65Gy < 17–20% was applied. Bladder limits varied widely (e.g., V70Gy < 5–35%, V65Gy < 25–30%, Dmax ≤ 80 Gy); Urethral limits were scarcely specified, with exceptions as D2% < 77 Gy, Dmax < 74.5–79.3 Gy, or D1% ≤ 79.3 Gy. Constraints in moderate hypofractionation regimens were less comparable due to dose heterogeneity, as shown in Table 2. Ultra hypofractionation regimens commonly set upper limits as Dmax or D0.03 cm^3^/D0.035 cm^3^ instead, limited to ≤ 35–41.2 Gy, < 42 Gy (or V42Gy < 0.03 cm^3^), and < 36.25–44 Gy in 5-fraction regimens for rectum, bladder, and urethra, respectively. One exception was Dmax of the urethra and bladder < 49.88 Gy, adapted to higher dose (50–55 Gy to IPLs) [67]. Two-fraction regimens applied limits in proportion to dose (e.g., 27/30 Gy or 26/32 Gy), such as rectal/urethral D0.03 cm^3^ < 27 Gy or urethral planning organ at risk volume (PRV) Dmax < 33.62 Gy [62]. Across fractionation schemes, a total of ten studies, most of which were ultra hypofractionation studies, established PRVs of 2–3 mm for OARs. Notably, only two studies reported OAR constraints for non-boost cohorts. One applied identical constraint definitions (e.g., rectal D0.03 cm^3^ < 105% of prescription) to both focal boost and non-boost regimens, with differences lying solely in prescription dose [61]. In the other, differing dose–fractionation schemes further precluded direct comparison [57].

Radiotherapy was delivered with the assistance of diverse guiding techniques and devices. Cone-Beam CT (CBCT) was the most frequently employed modality (22/42 studies), followed by kilovoltage (kV) imaging (standalone or CyberKnife-based), MRI-based adaptive workflows, portal imaging, and ultrasound. Image guidance was most commonly performed once daily prior to treatment, with some studies additionally acquiring both pre- and post-treatment images [62], [68]. Imaging guidance of higher frequency was reported in studies employing fiducial tracking with CyberKnife [51], [53], [74] and MRI-guided linear accelerator (MRI-Linac) adaptive workflows [57], [64], [65]. Conversely, the frequency of CBCT was reduced to once or twice a week in several cases, either after daily imaging of the first several fractions [29], [43], [44], [45], or when combined with a daily kV image [31]. Three MRI-Linac studies described online adaptive replanning workflows [57], [64], [65], while offline replanning was rarely referred to. Fig. S3 presents a qualitative comparison of PTV expansions across imaging modalities and fractionation schedules, with no obvious pattern visible.

Assistance markers for alignment included endogenous prostatic calcifications [29] and auxiliary devices, such as gold fiducials, rectal balloons [52], [65], as well as urethral catheters [31]. No specific differences were reported regarding image guidance strategies between focal boost and non-boost cohorts in any of the studies included.

Thirty-eight out of 42 studies prescribed androgen deprivation therapy (ADT) to patients, which typically spanned 6–12 months for intermediate-risk patients and was delivered in variable courses from one to 2–3 years for high-risk and very high-risk patients. Notably, the studies with no ADT use exclusively involved low- and intermediate-risk patients, except for one that included 27% high-risk patients [20]. No study explicitly differentiated ADT use between focal boost and non-boost cohorts.

Thirty-one studies reported clinical outcomes, while 11 did not. Conventional fractionation studies were generally longer (up to 10 years) than moderate or ultra hypofractionation (2–5 years). Clinical outcomes are summarized in Table 4 and Fig. S4, classified by fractionation regimens and standard vs focal boost schemes. With conventional fractionation, 5-year biochemical disease-free survival (bDFS) was 92–98.2% and declined to 68–90% at 10 years. Moderate hypofractionation studies reported 3-year bDFS ranging from 81% to 96% [45], [49] and a 5-year bDFS of 75.2% [39], added by combined biochemical/clinical progression-free survival (PFS) of 89.3% at 3 years [48] and 96.7% at 5 years [42]. In ultra hypofractionation studies, 5-year bDFS reached 93–100%, except for the cohort with a median 82-month follow-up, which resulted in a bDFS of 60.6% [53]. In conventional fractionation studies, the reported 5-year overall survival (OS) was 100% [28], whereas 10-year OS was 66–87.8%. The presently available data indicated OS rates of 88% at 3 years to 91.2% at 5 years for moderate regimens and 5-year OS of 86–100% for ultra hypofractionation.

**Table 4:** Clinical outcome by fractionation regimens.

		Conventional		Moderate hypo	Ultra-hypo	
		Standard	Focal boost	Focal boost	Standard	Focal boost
Biochemical disease-free survival, median (min, max) [N = number of studies]						
	3-year	−	95.5% (90.9–100%) [N = 2]	85.2% (81%–89.3^a^%) [N = 2]	−	100% [N = 1]
	5-year	85% [N = 2]	92% (92–93.2%) [N = 4]	86% (75.2–96.7^b^%) [N = 2]	100% [N = 1]	95% (93–100%) [N = 5]
	10-year	−	81% (68–90.1%) [N = 4]	−	−	−

Overall survival, median (min, max) [N = number of studies]						
	3-year	−	−	88% [N = 1]	−	−
	5-year	88% [N = 1]	100% [N = 1]	91.2% [N = 2]	−	94% (86–100%) [N = 3]
	10-year	−	69% (66–87.8%) [N = 3]	−	−	−

Footnotes:

a Including a 3-year biochemical/clinical relapse-free survival of 89.3% reported in Onjukka, E., 2017.

b Including a 5-year biochemical and clinical progression-free survival of 96.7% reported in Tree, A.C., 2023.

Four conventional fractionation studies compared focal boost with standard whole-gland radiotherapy and found non-inferior, sometimes superior, biochemical control results in focal boost arms. In the FLAME trial, the only prospective trial among them, the focal boost arm had a significantly higher 5-year bDFS and reduced risks of regional/distant metastasis [23], [24]. Similar outcomes were reported in a retrospective study in which a focal boost of 86 Gy to the IPLs resulted in higher 8-year bDFS, together with lowered rates of local, regional, and distant recurrence/metastasis, compared to 78 Gy whole-prostate irradiation [77]. Differences of OS/prostate cancer-specific survival (PCSS) between two regimes have not yet been observed with the available follow-up in both studies. The other two studies did not demonstrate a statistically significant gain in disease control from a focal boost: 80 Gy to IPLs added to 76 Gy for prostate ended in higher but non-significant 5-year bDFS (85% vs. 92%, p = 0.17) and OS (88% vs. 100%, p = 0.06) [28]. And 6-year bDFS rates were comparable between the two groups of 78 Gy to the prostate (± 82 Gy to IPLs) (85% ± 3% vs. 84% ± 3%) [33].

Beyond the aforementioned measures, changes in the IPL volume and ADC values on MRI were explored as imaging biomarkers for treatment response. Reduction or even complete disappearance of the IPLs was observed in two studies, with ADT administered to 32% [43] and 50% [22] of patients.

Clinician-reported adverse events (AEs) were graded with Common Terminology Criteria for Adverse Events (CTCAE) classification (in 31 studies) and Radiation Therapy Oncology Group (RTOG) classification (in nine studies), with classifications into acute and late genitourinary (GU)/gastrointestinal (GI) toxicities. Table 5 and Fig. S5 provides detailed toxicity profiles by fractionation regimens and schemes.

**Table 5:** Toxicity by fractionation regimens.

		Conventional		Moderate hypofractionation	Ultra-hypofractionation	
		Standard	Focal boost	Focal boost	Standard	Focal boost
Acute Toxicity, median (min, max) [N = number of studies]						
GU	≥ G2	46.2% (23.9–51%) [N = 3]	37.9% (13.3–56%) [N = 14]	36% (22.9–63.8%) [N = 9]	14.3% (10–36%) [N = 3]	20% (7.7–66.7%) [N = 19]
	≥ G3	5.0% [N = 1]	0% (0–7%) [N = 9]	4.3% (0–9%) [N = 5]	0% [N = 3]	0% (0–8%) [N = 19]
GI	≥ G2	10.1% (10–15.3%) [N = 3]	10.9% (0–45%) [N = 15]	15.3% (4.9–58.8%) [N = 9]	0% (0–4.8%) [N = 3]	6.4% (0–30%) [N = 18]
	≥ G3	0% [N = 1]	0% (0–5%) [N = 8]	0% (0–1.3%) [N = 7]	0% [N = 3]	0% (0–4%) [N = 18]

Late Toxicity, median (min, max)						
GU	≥ G2	23% (10.5–33%) [N = 3]	17% (0–44.6%) [N = 17]	30.1% (7.1–47%) [N = 9]	17.8% (7–28.6%) [N = 2]	18.4% (0–50%) [N = 14]
	≥ G3	5.8% (3.5–8%) [N = 2]	2.5% (0–6.8%) [N = 15]	2% (0–10%) [N = 9]	4.8% [N = 1]	0% (0–6%) [N = 12]
GI	≥ G2	12% (6.5–12.2%) [N = 3]	9% (0–21%) [N = 17]	17.8% (3.3–28%) [N = 9]	7.2% (0–14.3%) [N = 2]	6% (0–14%) [N = 14]
	≥ G3	1.7% (1.4–2%) [N = 2]	0% (0–5.4%) [N = 15]	3% (0–8%) [N = 9]	0% [N = 2]	0% (0–6%) [N = 11]

Footnotes:

G2, grade ≥ 2.

G3, grade ≥ 3.

GU, genitourinary toxicity.

GI, gastrointestinal toxicity.

Across regimens, grade ≥ 2 (≥ G2) GU events were generally more frequent than ≥ G2 GI events; acute toxicities were higher than late ones; and severe (≥ G3) events remained < 10%. In focal boost cohorts, ≥ G2 rates varied widely: acute GU 7.7–66.7% (median, 29.4%), acute GI 0–58.8% (median, 9.8%), late GU 0–50% (median, 20%), and late GI 0–28% (median, 9%). Ultra hypofractionation resulted in wider ranges of, but lower median levels of, toxicity rates compared to conventional and moderate hypofractionation. Comparison of toxicity profiles between focal boost and non-boost arms was conducted in some studies. In conventional-fractionation settings, focal boost did not increase toxicity, and one study showed lower ≥ G2 acute GU with focal boost (14.9% vs 23.9%, p = 0.03) [61]. In ultra hypofractionation, two studies suggested mild increases [57], [63] and one showed lower toxicity [78] with focal boost, with no valid statistical comparisons due to small sample sizes.

Patient-reported quality of life (QoL) was assessed in 14/19 of ultra hypofractionation studies. Among studies reporting QoL, four studies evaluated only urinary and bowel function, while 10 studies additionally assessed sexual/hormonal function, global health status or emotional function. Urinary or bowel function showed an initial decline followed by recovery in around 90% of studies, whereas sexual function more commonly demonstrated a decline followed by a sustained plateau (≈ 50% of studies).

To alleviate AEs, some conventional and moderate hypofractionation studies adopted urethral catheters (n = 5) to aid urethral delineation. Interventions implemented in ultra hypofractionation studies included urethrogram (n = 1) [68], prophylactic medications, such as alpha-1 blockers (n = 6) and baseline anti-diarrheas (n = 1 [61]), auxiliary devices (such as intrarectal immobilization tools, n = 3), and perirectal hydrogel spacers (n = 5).

## 4 Discussion

This scoping review summarizes current clinical evidence on focal boost EBRT to IPLs in prostate cancer. Focal boost approaches were generally feasible with acceptable toxicity, although substantial heterogeneity existed in imaging, target delineation, and dose-fractionation strategies. While the FLAME trial provides the strongest prospective evidence, most available data are derived from small or heterogeneous cohorts, highlighting the need for standardized prospective validation.

Consensus has not yet been reached regarding the optimal patient population for focal boost radiotherapy in prostate cancer. Growing evidence indicates that patients with high-risk localized disease derive the greatest benefit from focal dose escalation. The FLAME trial-derived model predicted an improvement of > 10% in 5-year bDFS for Grade Group 4–5 with a 95 Gy boost, while Grade Group 1 exceeded 90% bDFS at 77 Gy alone [78]. Similarly, an 86 Gy boost is an independent predictor of 5-year bDFS in Gleason Score 10 disease, according to a retrospective analysis [77], consistent with results favoring EBRT + ADT + brachytherapy boost for Grade Group 5 patients over EBRT with either ADT or prostatectomy [79]. Focal boost has also shown feasibility in the salvage setting after prostatectomy or brachytherapy, extending the application beyond localized prostate cancer [80], [81], [82].

Focal boost regimens, although largely based on the NCCN guidelines, have shown a trend toward higher per-fraction dose in fewer total fractions, and lower whole-gland dose, as shown in Table S1. For conventional fractionation, a validated SIB paradigm of 2.2 (2.7 to IPLs) Gy 35 fractions was established by the FLAME trial [24]. More intensely elevated per-fraction doses are administered in moderate (> 3.5 Gy to the whole gland) and ultra hypofractionation regimens (> 10 Gy to the whole gland), as well as one spatially fractionated IPLs boost (12–14 Gy) after standard conventional irradiation [25]. However, these high per-fraction doses may reduce the safety margin between ideal tumor dose delivery and normal tissue tolerance, making success contingent on high-fidelity image guidance, motion control, and a stringent urethral/rectal dose cap. In this context, Westley et al.’s work might have provided an illustrative example [57]. In their two-fraction MR-Linac regimen, each fraction (13.5 Gy to IPLs) was delivered in two subfractions on the same day, with the second subfraction administered only after readjustments were made to the plan. Similarly, advanced MR-guided adaptive workflows may address these challenges at the technical level. The online adapt-to-shape and adapt-to-position strategies, combined with real-time motion monitoring and conditional treatment interruption with completion replanning, ensure accurate delivery of high doses. Focusing more on toxicities, whole-gland dose was decreased to 7–7.25 Gy in 5 fractions in five ultra hypofractionation regimens, as compared to the preferred 7.25–8 Gy in 5 fractions by NCCN guidelines [3]. Conceptually, this “boost-up/de-escalate-down” strategy trades homogeneous coverage for biological intensification where it matters most (the IPLs), and is suggested to be able to preserve, or even improve, toxicity profiles when planning is driven by normal-tissue constraints [52], [58], [59], [65], [68]. Taken together, these observations suggest a conceptual planning approach: escalation of IPL EQD2 may be pursued where anatomically feasible, while whole-gland dose de-escalation may be considered when constrained by proximity to OARs.

Since focal escalation concentrates dose within milliliter-scale subvolumes, reproducible IPL delineation becomes a primary determinant of both efficacy and toxicity. However, standardized criteria for imaging modality selection (e.g., MRI, PET, or multimodality approaches) and contouring strategies (e.g., PI-RADS thresholds, PET uptake thresholds, or biopsy confirmation) remain lacking across studies. On mpMRI, although PI-RADS 4–5 lesions are arguably considered as IPLs to be boosted, debate exists over the management of PI-RADS 1–3 lesions, which were included after biopsy confirmation in more proactive schemes [31]. This demands judicious weighing of the cost of the invasive diagnostic technique and the risks of recurrence. An increasing number of studies are utilizing PET/CT for IPL delineation for its high sensitivity, especially in localized high-risk [83] and recurrent [84] prostate cancer [85]. The identification of IPLs on PET/CT is more study-specific, varying from visual contouring to automatic contouring based on absolute SUV or %SUV thresholds (commonly 20–60%) [50]. Emmett et al. proposed a structured ^68^Ga-PSMA PET-based interpretation framework integrating uptake pattern and intensity, and showed its better diagnostic performance than mpMRI (sensitivity 88% vs 83%; specificity 64% vs 53%), which may help address the lack of standardized PET/CT-guided IPL identification protocols [86]. Yet, given variable specificity [87], [88], PET/CT is currently best used to complement mpMRI. Hybrid plans of mpMRI and PET/CT often escalate overlapping regions and de-escalate discordant areas [50]. Emerging options, such as PET/MRI [89], restriction spectrum MRI [90], and prostate-cancer-specific MRI by genetic amplified nanoparticles [91], are promising but still experimental.

Several studies limited boosting to only 1–3 IPLs, citing weak concordance between the small size of non-dominant foci and pathology [92]. For instance, in Pollack et al., the second/third largest IPLs averaged < 1 cm^3^ versus 2.5 cm^3^ of the dominant lesion [25]. Most studies, on the other hand, escalated the doses of all visible IPLs. Pragmatically, only lesions with strong multiparametric concordance (mpMRI ± PSMA-PET) and adequate size may warrant focal boost; for smaller or discordant lesions, case-by-case decisions need to be made, until head-to-head trials of “dominant-only” vs “all-visible” boosting are available.

The initiation of ADT also has a significant influence on imaging-based IPL delineation. Neoadjuvant ADT improves outcomes in intermediate–/high-risk disease [93] but compromises lesion conspicuity. Lesions show increased ADC and decreased perfusion after ADT on MRI [94], as well as reduced PSMA or choline uptake and thus lowered SUVmax on PET/CT [95]. Whilst some IPLs can still be identified post-ADT on MRI [94] and PET/CT [35], concerns are growing about the potentially impaired delineation accuracy. Thus, contouring IPLs based on pre-treatment imaging serves as a solution widely used in focal boost protocols. Postponing ADT until after radiotherapy initiation (i.e., concurrent/adjuvant ADT) is an alternative, with better OS results than neoadjuvant/concurrent ADT in prostate-only radiotherapy according to a *meta*-analysis [96]. Advanced methods may overcome post-treatment obscuration by capturing non-morphological tumor features. For instance, MRI texture analysis can capture residual microstructural heterogeneity despite signal attenuation [97], while ^68^Ga-PSMA PET/CT reflects tumor-specific molecular activity, and shows superior concordance with pathology compared with mpMRI [98].

As radiotherapy now permits true dose sculpting, “dose painting by numbers” departs from contour-based boosting by prescribing voxel-level doses informed by imaging-derived biology [99]. Thorwarth et al. integrated multiparametric PET/CT and DW-MRI to generate tumor-probability maps, delivering 74 Gy to the whole prostate with a median 75.7 Gy (72.5–80 Gy) boost to high-risk subvolumes [100]. Population-level cell-density and tumor-probability atlases have also been fused with individual anatomy to yield tumor control probability (TCP)-optimized prescriptions [101], [102]. An ongoing trial (NCT03658434) blends concepts of dose painting by both numbers and contours, wherein manually contoured IPLs receive 5 Gy in 4 fractions, meanwhile subvolumes identified by low ADC values are boosted to 7 Gy/fraction [103]. Less reliant on manual margins, such voxel-level planning may be particularly useful after neoadjuvant ADT.

Most studies used guideline-concordant ADT, which lasted 4–6 months for intermediate-risk patients, and 2–3 years for high-risk cohorts. The course of ADT was shortened to six months tentatively in some focal boost schemes, assuming improved local control from focal boost might compensate for the reduced systemic therapy [50]. Nonetheless, a retrospective analysis showed ADT conferred benefit in intermediate-risk disease irrespective of focal boost, suggesting its indispensable role [104]. The treatment reaction of combined focal boost SBRT with shorter ADT in high-risk patients is being examined by an ongoing trial (HYPOPRIME, NCT06204341). Until the non-inferiority of abbreviated ADT with focal boost is supported by more solid evidence, ADT duration should adhere to risk-specific standards.

Adding margins to PTV is more of a compensation for setup error and unmodeled motion than a treatment goal. In ultra hypofractionation, minor geometric misses can precipitate major biological underdose. Therefore, an optimal margin cannot be defined without the context of imaging frequency and inter-fraction motion control. Posterior margin reduction (or a 2 mm low-dose buffer PRV) should be conditional on stable bowel/bladder preparation and online correction; otherwise, rectal sparing may come at the expense of inadequate target coverage. Advanced guidance, such as electromagnetic tracking catheters [105], [106] and real-time adaptive delivery [107], can justify tighter margins and may lower toxicity, but should be paired with explicit stop-treat thresholds and dose reconstruction to the IPLs.

Promising oncological outcomes with focal boost strategies have been shown in studies of conventional and ultra hypofractionation regimens, with 5-year bDFS rates generally exceeding 90%. Results after moderate hypofractionation studies are inconsistent: single-arm ones yielded mixed results, while the only comparative study (DELINEATE) found comparable results in cohorts treated with moderate hypofractionated and conventional fractionated focal boost radiotherapy [42]. In conventional fractionation studies, focal boost was associated with either superior or non-inferior outcomes regarding bDFS and recurrence rates, endorsed by the FLAME trial [24]. Such direct comparisons of oncological outcomes between focal boost and non-boost approaches remain scarce in moderate and ultra hypofractionation settings. Moreover, data on OS and PCSS are sparse, leaving it unclear whether gains in local control with focal boost can translate into long-term survival benefits. However, this to some extent reflects insufficient short follow-up rather than a lack of efficacy, highlighting the need for continued longitudinal reporting of present studies. Nonetheless, it should be noted the role of patient selection as a critical confounder. Available long-term data generally come from a substantial proportion of low- or intermediate-risk patients, who may already be adequately treated with standard whole-gland doses. In a retrospective study, the 8-year PCSS surpassed 90% in both groups receiving 78 Gy with/without a focal boost, each consisting of more than 35% of patients at intermediate risk [18]. On the other hand, evidence supports that a brachytherapy boost can add benefits to therapies of EBRT + ADT regarding 5-year PCSS and distant metastasis-free survival in patients with Gleason Grade Group 5 [79]. These findings collectively suggest that the greatest benefit of focal boost may lie in selected high-grade populations. Other outcome measures, such as early IPL shrinkage or ADC rise, may show potential as early-response prognostic biomarkers with analyses explicitly adjusting for ADT-related effects on imaging signals.

Clinician-reported AEs after focal boost EBRT show a consistent overall profile: most cohorts keep ≥ G2 events below 40%, and ≥ G3 remains uncommon. Head-to-head and cross-study comparisons do not manifest systematically higher toxicity with focal boost compared to non-boost regimens. Apparent discrepancies across studies should be partly attributable to methodological variations in acute/late cut-offs, grading scales (RTOG vs. CTCAE) which may alter reported rates by up to 10% [42], [55], [108], crude vs. actuarial statistics, and baseline symptoms. Planning parameters and anatomical factors have an impact on spatial dose distribution, thereby affecting toxicity. The FLAME trial demonstrated a dose–response relationship for late GU toxicity, emphasizing the importance of limiting urethral/bladder hot spots [109]. Small-volume, high-dose metrics (e.g., D0.03 cm^3^/Dmax) for rectum and bladder are particularly predictive in hypofractionated focal boost regimens [110], [111], and the IPL proximity to the urethra further modulates risk [26], [53]. Geometry and motion management interact with these constraints: posterior margin reduction and electromagnetic tracking integrated with real-time adaptive workflows enable tighter PTVs without compromising toxicity control [105], [106]. The fractionation schedule is another lever. Shortening overall treatment time risks elevating toxicity while enhancing tumor control. In Hypo-FLAME analyses, once-weekly ultra hypofractionation yielded fewer GU events than twice-weekly schedules [52], [65]. Patient-level factors, including age, rectal volume, and prior pelvic surgery, remain strong non-modifiable predictors of toxicity and should be a focus of guide counseling and mitigation strategies [112], [113], [114]. On the mitigation side, rectal hydrogel spacers reduce rectal dose and GI events [115], [116], especially in high-dose SBRT, though the routine use in standard-dose regimens remains controversial [59]. Future research priorities include randomized evaluation of delivery cadence, clearer indications for spacers, and a harmonized AE reporting system considering patients-reported QoL/MCIC to align toxicity readouts with what patients feel and clinicians can act on.

Admittedly, there are several limitations in our work. First, the primary search was conducted in PubMed. Although reference lists and relevant reviews were screened to reduce omissions, eligible studies indexed exclusively in other databases may have been missed. The literature search was conducted up to April 2025. While additional studies may have been published since then, this reflects the inherent time lag in evidence synthesis, and the present review captures the most comprehensive body of evidence available at the time of analysis. Additionally, the predominance of retrospective and small-sample studies in this emerging field constrained this review. Most prospective evidence derives from phase I/II trials with limited accrual and relatively short follow-up, and larger cohorts with longer observation are typically retrospective. Together, these features introduce substantial heterogeneity. In addition, several reports did not present outcomes and toxicity stratified by focal boost versus non-boost arms, militating against cross-study comparisons and precluding robust quantitative synthesis. Consequently, our conclusions should be interpreted with caution. Yet, this does not undermine the positive meaning of this work to give a systematic, updated summary of published clinical studies in this field, facilitating further exploration.

Current evidence suggests that focal boost EBRT is feasible and may improve biochemical control without increasing toxicity when constraint-driven planning is applied, especially in high-risk prostate cancer. However, boost dose/fractionation, IPL delineation workflows, and constraint sets (particularly small-volume OAR limits such as D0.03 cm^3^/Dmax) remain heterogeneous, and long-term survival benefit is unproven. Beyond large prospective trials with standardized endpoints, priority should be given to pragmatic, multi-institutional prospective studies that harmonize contouring and mandate core dose/toxicity reporting, including patient-reported outcomes, to define how to best deliver focal boost in routine practice.

## CRediT authorship contribution statement

**Ying Liu:** Writing – review & editing, Writing – original draft, Visualization, Project administration, Methodology, Investigation, Formal analysis, Data curation, Conceptualization. **Shangbin Qin:** Writing – original draft, Methodology, Investigation, Formal analysis, Data curation. **Xueying Ren:** Writing – original draft, Methodology, Investigation, Formal analysis, Data curation. **Yun Bai:** Writing – review & editing, Conceptualization. **Xianshu Gao:** Writing – review & editing. **Hongzhen Li:** Writing – review & editing, Funding acquisition. **Mingwei Ma:** Writing – review & editing, Writing – original draft, Methodology, Investigation, Funding acquisition, Formal analysis, Data curation, Conceptualization.

## Funding

This work was supported by Beijing Natural Science Foundation [grant number L232126]; Capital Health Development Research Programme [grant number 2024-2-4075]; Peking University Teng Yun Clinical Research Special Project [grant number TY2025001]; Central High-level Hospital Clinical Research Programme [grant number 2024CX21]; and Peking University Clinical Scientist Training Program [grant number BMU2025PYJH033].

## Declaration of competing interest

The authors declare that they have no known competing financial interests or personal relationships that could have appeared to influence the work reported in this paper.

## Appendix A Supplementary data

Supplementary Data 1 Detailed literature search strategy; additional figures illustrating EQD2 for IPL boost doses, PTV expansion margins across image guidance modalities and fractionation schedules, bDFS over follow-up according to fractionation regimen and focal boost use, and ≥ G2 toxicity (acute and late) stratified by fractionation; and a summary table of focal boost dose/fraction regimens referenced to NCCN guidelines.
