# Supplementary material for: External beam focal boost radiotherapy to intraprostatic lesions in prostate cancer: a scoping review
Source: Phys Imaging Radiat Oncol. 2026 Apr 25;39:100980. doi: 10.1016/j.phro.2026.100980 (PMC13140468; doi:10.1016/j.phro.2026.100980)
Supplement: Supplementary Data 1 — Detailed literature search strategy; additional figures illustrating EQD2 for IPL boost doses, PTV expansion margins across image guidance modalities and fractionation schedules, bDFS over follow-up according to fractionation regimen and focal boost use, and ≥ G2 toxicity (acute and late) stratified by fractionation; and a summary table of focal boost dose/fraction regimens referenced to NCCN guidelines. [file mmc1.pdf]

Supplementary Material

Catalogue

Supplementary Material A. Full literature searching strategy 2
Figure S2. PTV expansion by fractionation. 4
Figure S3. PTV expansion margins across different image guidance modalities and fractionation schedules. 5
Figure S4. Reported bDFS over follow-up time according to fractionation regimen and use of focal boost. 6
Figure S5. ≥ Grade 2 toxicity. 7
Table S1-Dose/Fraction of Focal Boost Regimens as referred to the NCCN Guidelines[1] 8

Abbreviation

EQD2, equivalent dose in 2 Gy fractions
bDFS, biochemical disease-free survival
IPL, intraprostatic lesion
PTV, planning target volume

Supplementary Material A

The literature searching strategy used in this systematic review was as follows:

(((focal boost) OR (boost) OR (dose escalation) OR (dose-escalated) OR (concomitant boost) OR (micro-boost) OR (intensification)) AND ((intraprostatic lesion) OR (intra-prostatic lesion) OR (intraprostatic nodule) OR (intraprostatic tumor) OR (dominant intraprostatic nodule) OR (dominant intraprostatic lesion) OR (intraprostatic) OR (IDN) OR (dominant) OR (IPL) OR (IPN) OR (DIL) OR (focal lesion) OR (macroscopic lesion)) OR (simultaneous integrated boost) OR (dose painting)) AND (((Prostate) OR (prostate cancer)))

*To minimize the risk of missing relevant studies, a deliberately sensitive search strategy was adopted. Boost-related terms (e.g., “simultaneous integrated boost” and “dose painting”) were not strictly restricted to intraprostatic lesion terminology at the search stage, as terminology varies across the literature. Eligibility was subsequently determined through full-text screening to ensure inclusion of studies delivering focal boost to intraprostatic lesions.

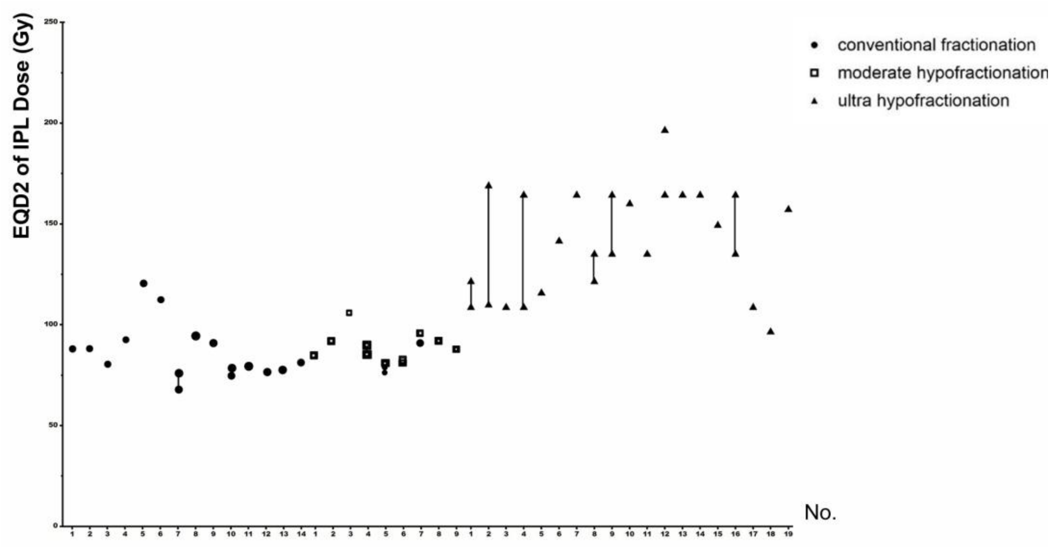

Figure S1. EQD2 for IPL boost doses across included studies.

Each symbol represents a study, and error bars indicate the reported EQD2 range for the IPL. Studies are arranged along the x-axis according to the order presented in Tables 1–3: conventional fractionation (Table 1), followed by moderate hypofractionation (Table 2), and ultra hypofractionation (Table 3).

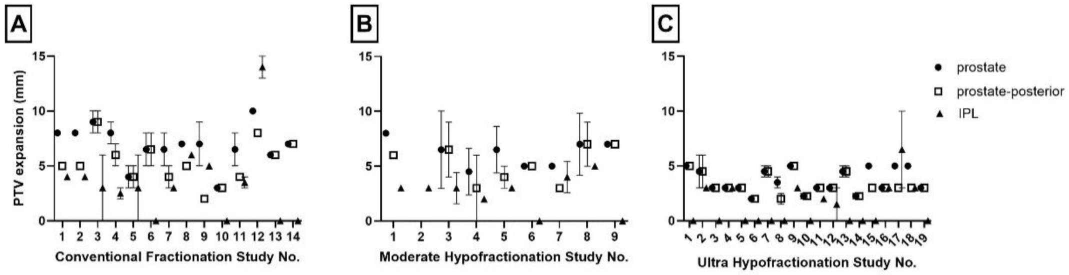

Figure S2. PTV expansion by fractionation.

Panels show conventional (A), moderate (B), and ultra-hypofractionation (C) regimens. The x-axis represents study number (as in Tables 1–3). Error bars indicate the reported PTV expansion range.

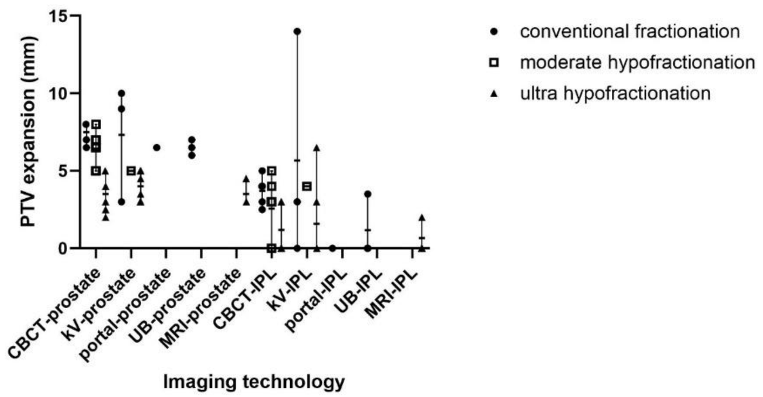

Figure S3. PTV expansion margins across different image guidance modalities and fractionation schedules.

Each data point represents one study. The x-axis denotes the image guidance modality used for treatment delivery, and the y-axis shows the corresponding PTV expansion (mm). Prostate and IPL margins are presented separately. Different symbols indicate fractionation schedules (conventional fractionation, moderate hypofractionation, and ultra-hypofractionation). When a study reported PTV expansion as a range, the midpoint value was plotted. These plots are intended for qualitative visualization of reported margins across imaging techniques rather than for formal statistical comparison.

Abbreviation:

CBCT, cone-beam computed tomography
portal, portal X- ray
kV, kilo voltage imaging
UB, ultrasound
MRI, magnetic resonance imaging

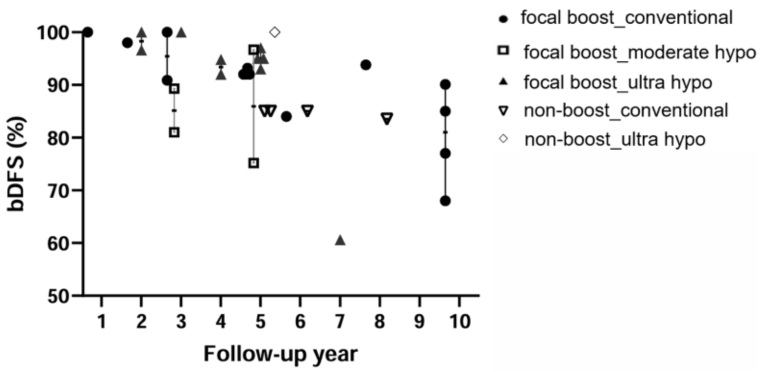

Figure S4. Reported bDFS over follow-up time according to fractionation regimen and use of focal boost.

Scatter plots present bDFS values reported at discrete follow-up time points across included studies. Each dot represents a single study reporting bDFS at a given follow-up year. Rows indicate yearly follow-up intervals. No non-boost cohort using moderate hypofractionation was identified.

This figure is descriptive and does not imply longitudinal trends or comparative effectiveness.

Abbreviation:

conventional, conventional fractionation cohort
moderate hypo, moderate hypofractionation cohort
ultra hypo, ultra hypofractionation cohort

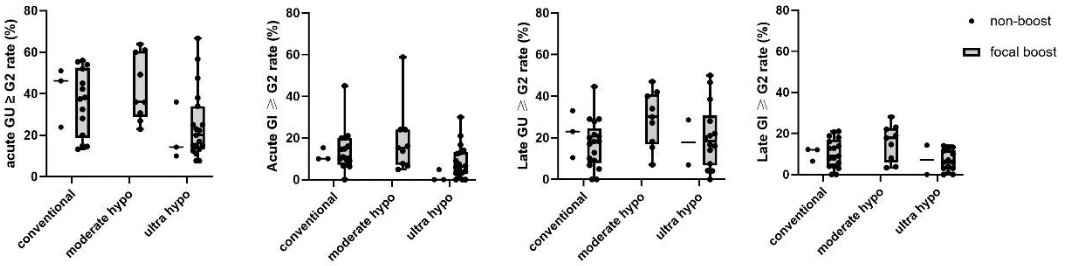

Figure S5. ≥ Grade 2 toxicity.

Non-boost cohorts are shown as individual points due to small sample numbers; focal boost cohorts are presented as boxplots to illustrate distribution. Symbols correspond to genitourinary (GU) and gastrointestinal (GI) toxicity, and error bars indicate the reported range when available.

Abbreviation:

conventional, conventional fractionation cohort
moderate hypo, moderate hypofractionation cohort
ultra hypo, ultra hypofractionation cohort

Table S1-Dose/Fraction of Focal Boost Regimens as referred to the NCCN Guidelines[1]

EBRT Regimen (definitive treatment)	Preferred Dose/Fraction by NCCN Guidelines [3]	Focal Boost Regimens								
		a) of whole gland dosage following the NCCN Guidelines [3]			b) of fewer fractions with higher per-fraction dose			c) of de-escalated whole-gland dose		
Conventional	1.8–2 Gy × 37–45 fx[2]	whole-gland	1.8–2 Gy	× 38–44 fx	whole-gland	2.02–2.35 Gy	× 32–38 fx			
		IPL	1.88–2.26 Gy		IPL	2.12–2.71 Gy				
		whole-gland	1.8–2 Gy	× 38 fx						
		IPL	12–14 Gy	× 1 fx						
Moderate Hypofractionation	2.5–3 Gy × 20–28 fx	whole-gland	3 Gy	× 20 fx	whole-gland	3.5–3.6 Gy	× 15–16 fx			
		IPL	3.1–3.75 Gy		IPL	3.56–3.8 Gy				
Ultra Hypofractionation	9.5 Gy × 4 fx<br>7.25–8 Gy × 5 fx<br>6 Gy × 6 fx<br>6.1 Gy × 7 fx	whole-gland	7.25–8 Gy	× 5 fx	whole-gland	12–13.5 Gy	× 2 fx	whole-gland	7–7.25 Gy	× 5 fx
		IPL	8–10 Gy		IPL	13.5–6 Gy		IPL	8–10 Gy	
		whole-gland	9.5 Gy	× 4 fx						
		IPL	11 Gy							

Abbreviation:

[1] the NCCN guidelines: the National Comprehensive Cancer Network. Prostate Cancer, Version 2. 2026. NCCN Clinical Practice Guidelines in Oncology https://www.nccn.org/professionals/physician_gls/pdf/prostate.pdf.

[2] fx: fraction(s)
